# Role of extensification strategies in chicken meat quality

**DOI:** 10.1016/j.psj.2025.105717

**Published:** 2025-08-28

**Authors:** Agnieszka Ludwiczak, Patryk Sztandarski, Joanna Składanowska-Baryza, Karolina Szulc, Gabriela Cieleń, Aneta Jaszczyk, Magdalena Solka, Grzegorz Pogorzelski, Jarosław O. Horbańczuk, Joanna Marchewka, Ewa Sell-Kubiak

**Affiliations:** a Department of Animal Breeding and Product Quality Assessment, Poznań University of Life Sciences, Słoneczna 1, Suchy Las, 62-002, Poland; b Institute of Genetics and Animal Biotechnology of the Polish Academy of Sciences, Jastrzębiec, 05-552, Magdalenka, Poland; c Department of Genetics and Animal Breeding, Poznań University of Life Sciences, Wołyńska 33, Poznań, 60-637, Poland

**Keywords:** Perch, Lucerne, Poultry meat, Stocking density, Myopathy

## Abstract

The latest studies suggest a beneficial influence of husbandry factors, including increased space allowance, access to perches, and roughage, on the welfare of chickens; however, their effects on meat quality are divergent. Two experiments (EXP1 and EXP2) were performed to determine the husbandry factors affecting the meat quality of 42-day-old Ross 308 chickens (*n* = 180). The examined factors in EXP1 were stocking density (35 kg/m2 vs 41 kg/m2) and enrichment (access to perches), whereas in EXP2, enrichment (perches) and access to roughage (dried lucerne) were examined. The meat quality examination included physicochemical traits and myopathy incidence and severity assessment. In EXP1, enrichment did not affect fresh meat quality traits, whereas stocking density influenced the b* index and post-thaw pH, with birds kept at lower stocking density showing the yellowest meat. Enrichment significantly affected proximate composition, as perch access increased moisture content and reduced fat content, while higher stocking density elevated fat levels. In EXP2, enrichment did not affect fresh meat quality, but roughage provision altered pH24, pH48, and b* values, with strong interactions between factors. Freezer storage analyses revealed enrichment effects on thaw and cooking loss, Warner-Bratzler and BMORS textural traits, as well as moisture and fat composition. Birds with perch access exhibited higher moisture and lower fat content, alongside reduced Warner-Bratzler force and energy (increased tenderness). Roughage also modulated pH and color traits, with broilers showing the yellowest meat. To conclude, stocking density had a limited influence on the overall meat quality. Enrichment with perches in combination with roughage causes the most significant changes in chicken meat attributes. It increased moisture and reduced fat content in meat, while also enhancing tenderness after storage when combined with access to roughage. Roughage provision improved pH stability and reduced excessive yellowness of meat.

## Introduction

Poultry meat remains the most widely consumed type worldwide, with global production exceeding 146 million tons in 2023, of which approximately 16 % originated from the European Union (FAO, 2024). In recent years, consumer expectations regarding animal-derived foods have evolved considerably. Increasingly, consumers are paying attention not only to the nutritional value and safety of products, but also to the source of animal products and the welfare conditions under which animals are raised. Surveys, reports, and market studies consistently demonstrate a growing interest in higher rearing welfare standards for farm animals, as many consumers associate improved welfare with superior product quality (Albergamo et al., 2022; European Commission, 2023). This shift in consumer attitudes has motivated researchers and producers to investigate housing strategies that could enhance both animal well-being and meat quality.

### Enrichments encouraging natural behaviors and physical activity

One approach to improving welfare is using environmental enrichment—modifications to the birds’ living environment that encourage natural behaviours and physical activity. Various enrichment types can be implemented, each provoking different behavioral responses (Riber et al., 2018). In the present study, we focused on the provision of perches. Perching is a natural behaviour for chickens, and its facilitation in intensive systems can improve leg health, reduce lameness, and strengthen bones and muscles (Aksit et al., 2017; Bailie et al., 2013; Jacobs et al., 2023). While the benefits of enrichment for bird welfare are well documented, its influence on meat quality remains unclear. The literature reports considerable discrepancies in the effects of enrichment on key meat characteristics, highlighting the need for further research (Marchewka et al., 2023). The available research results indicate that access to perches does not influence the water holding capacity of meat, as measured by pressure, temperature, and gravitation (Fidan et al., 2020; Kiyma et al., 2016). The color of chicken meat varied, as the access to perches led to decreased L* value (Aksit et al., 2017; Fidan et al., 2020). None of the studies examined whether perches may influence meat texture or the incidence of myopathies. Notably, most existing studies have explored the impact of outdoor access or free-range systems. At the same time, fewer have examined the influence of indoor enrichment methods—such as straw bales or perches—on specific meat quality traits.

### Nutritional enrichments

Another welfare-oriented practice relevant to meat production is access to roughage to encourage foraging behavior and reduce hunger and frustration (Tahamtani et al., 2025). Although excessive dietary fibre can impair nutrient digestibility in poultry, providing an appropriate type and quantity can support gastrointestinal tract development and improve digestive physiology (Jha and Mishra, 2021). The effect of roughage access on physicochemical meat quality is underlined by the variation in water-holding capacity measures (like drip loss), and meat yellowness (He et al., 2021). However, the influence of roughage access on elemental chemical composition is inconsistent. For example, Zheng et al. (2019) stated no effect of lucerne supplementation on the content of crude protein, crude fat, and dry matter in chicken meat (breast and leg), while He et al. (2021) pointed out that a certain level of supplementation may decrease the crude protein content in chicken compared to the control group (no dried lucerne in the diet).

### Space allowance

A further extensification measure is the reduction in stocking density, which directly influences welfare and productivity. High stocking densities can restrict movement, increase competition for feed and water, elevate stress levels, and exacerbate health issues such as footpad dermatitis (Nasr et al., 2021; Sugiharto, 2022). Conversely, lower stocking densities can promote greater mobility and more natural behaviour, which may indirectly affect meat quality by altering muscle development and fat deposition (Nasr et al., 2021). Given that physical activity has been linked to changes in meat texture, color, and composition, stocking density is a critical variable in meat quality research. The amount of activity has proven to be one of the factors defining the proportion of slow-twitch oxidative and fast-twitch glycolytic fibers (Listrat et al., 2016). The share of these fibers influences the color and tenderness of meat, with a higher proportion of oxidative fibers resulting in redder color (high content of myoglobin) and lower shear force (smaller fiber size, higher fiber density) (Lee et al., 2016).

Against this background, we hypothesized that these measures, individually and in combination, would improve selected aspects of meat quality by promoting higher activity levels, enhancing muscle development, and supporting digestive health. Thus, the present study aimed to evaluate the combined effects of three extensification strategies—environmental enrichment through perch provision, increased space allowance via reduced stocking density, and dietary supplementation with roughage in the form of dried lucerne—on the meat quality of Ross 308 broiler chickens.

## Materials and methods

The study was conducted as part of the mEATquality project, funded by the European Union’s Horizon 2020 Research and Innovation Program under Grant Agreement No 101000344.

### Ethics approval

The experiment described in this study did not require approval from the Polish Ethical Commission, as it fell outside the scope of interventions requiring ethical review under the Act of 15 January 2015 on the Protection of Animals Used for Scientific and Educational Purposes (Journal of Laws of the Republic of Poland, Item 266, Article 1). The study replicated standard on-farm production cycles and involved no invasive procedures or interventions beyond routine husbandry. All practices adhered strictly to typical broiler management protocols, complied with relevant national legislation, and established best farming practices. Additionally, the entire study was carried out in strict accordance with the principles and specific guidelines outlined in the Guide for the Care and Use of Agricultural Animals in Research and Teaching, 4th edition (2020).

### Experimental animals, groups, and housing conditions

The experiment was conducted on the farm of the Institute of Genetics and Animal Biotechnology, Polish Academy of Sciences, located in the Mazovian region of Poland (52.032404, 20.826490), between June and September 2023.

### Birds and the experimental design

Day-old Ross-308 chicks (*n* = 180) of both sexes were used in the study. The average weight at placement was 57.0 g ± 1.0 (mean ± SD). As presented in the experimental design in Table 1, the chicks were assigned to six treatment groups, with two fixed factors per experiment, which were stocking density (DEN; with two levels 35kg/m^2^ –DEN-35 and 41kg/m^2^ –DEN-41) and access to perches (EN; with two levels no enrichment –N-PER and enrichment via perches PER) in experiment one (EXP1), whereas access to perches (same as in EXP1) and to roughage (RO; with two levels no roughage –N-LU and access to Lucerne - LU) in experiment two (EXP2). Two of the treatment groups, group 1 and group 2, were common in both experiments. Each group contained five replications of the treatment group, with six birds in each replication, resulting in 30 birds per group. Treatment groups with access to roughage were given dried lucerne bales (1400 g), offered in two hanging nets per pen. The nets were replaced with fresh dried lucerne every 7 days, allowing the chickens to consume additional roughage through pecking and foraging behavior regularly (EFSA , 2023). The enriched pens were equipped with a 0.6-meter-long perch with two perching levels (10 cm and 30 cm). The perches (50 × 50 mm) had rounded edges.

**Table 1:** Experimental design.

Treatment groups	Stocking density (DEN)	Enrichments (EN)	Roughage (RO)	EXP 2 EXP1
Group 3 *n* = 30	35kg/m^2^ (DEN-35)	No enrichment (N-PER)	No roughage (N-LU)	
Group 4 *n* = 30	35kg/m^2^ (DEN-35)	Environmental enrichment: perches (PER)	No roughage (N-LU)	
Group 1 *n* = 30	41kg/m^2^ (DEN-41)	No enrichment (N-PER)	No roughage (N-LU)	
Group 2 *n* = 30	41kg/m^2^ (DEN-41)	Environmental enrichment: perches (PER)	No roughage (N-LU)	
Group 5 *n* = 30	41kg/m^2^ (DEN-41)	No enrichment (N-PER)	Roughage: dried lucerne (LU)	
Group 6 *n* = 30	41kg/m^2^ (DEN-41)	Environmental enrichment: perches (PER)	Roughage: dried lucerne (LU)	

### Basic housing conditions and feeding

The birds were kept in a building with controlled microclimate, in pens with sawdust bedding (0.65 × 0.65m^2^, i.e., 0.42m^2^ area each). One and two-day-old chicks were kept under continuous lighting (24 hours of light) to promote early feed intake and adaptation. Three-day-old and older birds were reared under a photoperiod of 15 hours of natural light and 9 hours of darkness. Natural light was provided through uncovered windows (the ratio of window area to floor area was 1:7). The pens were equipped with a temperature‒humidity monitoring system. The temperature was set at 34°C at the beginning of the experimental period (day 0 of age) and was gradually decreased to a temperature of 19-21°C with a constant relative humidity of 45 %. Feed and water were provided *ad libitum*. Broiler chicks were vaccinated against Marek’s disease and respiratory pathogens such as infectious bronchitis virus (IBV) and Newcastle disease virus (NDV), using spray or eye-drop application at the hatchery. The birds were fed a conventional diet consisting of corn, wheat, soybean meal, sunflower seeds, dehulled sunflower seeds, potato processing products, animal fat (poultry), vegetable oils and fats (sunflower-crude), wheat bran, and vegetable oils and fats (sunflower seeds). The feed chemical composition, feeding schedule, and feed ingredients are provided in Table 3, Table 4. The feed intake in the feeding phases (starter, grower 1, grower 2, and finisher) is given in Table 5. The feed intake was measured daily by weighing the feed provided and the leftover feed in each pen. The average feed intake per bird per day was calculated, and the cumulative feed intake for each phase was summarized.

### Meat quality examination

At the age of 41 days, the birds were slaughtered by trained slaughter personnel via cervical dislocation and bleeding. After the carcasses were chilled for 12 hours, the left and right *pectoralis major* muscles were cut from each carcass. The muscles were individually packed in plastic boxes and transported to the laboratory for meat quality analysis in a meat truck at 2-5°C temperature.

**Fig. 1:**
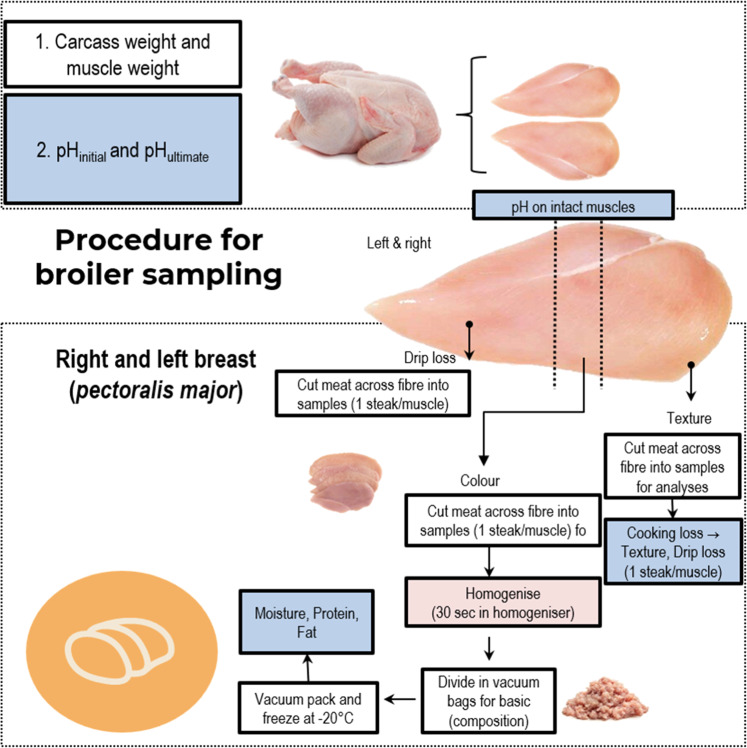
Procedure and timeline for chicken broiler meat sampling.

Each muscle was cut into upper, middle, and lower parts, as shown in Fig. 1. The number of samples per treatment group and repetitions of measurements are presented in Table 2. The upper part was subjected to pH measurement, weighed, packed in a low vacuum, and frozen (-20°C). The middle parts were used for color and EZ-Drip Loss measurements, whereas the lower parts were used for basic chemical composition examination. After two months of storage the frozen samples were thawed at 4°C for 24 h. The following measurements were made on the freeze-stored samples: for thaw loss, pH, cooking loss, and texture. The thaw loss was calculated from the weight difference before and after frozen storage. The pH was measured using a glass-calomel electrode (Lo 406- M6-DXK-S7/25, Mettler Toledo, Columbus, OH, USA) connected to a temperature-compensated pH meter (type 1140, Mettler Toledo, Columbus, OH, USA). The color was measured with a CM 700d spectrophotometer (Konica Minolta, Amsterdam, The Netherlands). The shear force measurements were made with the Warner-Bratzler V-shaped blade with the TA.XT Plus Texture Analyser (Stable Micro Systems, Warrington, UK). The basic chemical composition was examined according to AOAC (Association of Official Agricultural Chemists (AOAC), 2007). The methodologies used for pH, color, thaw loss, Warner–Bratzler texture measurement (WBSF), and basic chemical composition examination were described by Ludwiczak et al. (2024).

**Table 2:** Meat quality measures.

Traits	Samples per group	Measures per sample
pH45	*n* = 30	3
pH24	*n* = 30	5
pH48	*n* = 30	5
pH (thawed)	*n* = 30	5
Colour (L*,a*, b*)	*n* = 30	6
EZ-Drip Loss %	*n* = 30	2
Thaw loss %	*n* = 30	1
Cooking loss %	*n* = 30	2
Warner Bratzler	*n* = 30	5
BMORS	*n* = 30	6

**Table 3:** Feed composition and feeding schedule.

		Starter	Grower 1	Grower 2	Finisher
Feeding schedule (days)	0-9	10-20	21-32	>32	
	Energy (kcal/kg)	3155.00	3100.00	3145.00	3240.00
Feed analytical ingredients	Crude protein %	21.30	19.90	18.7	18.6
	Raw ash %	5.50	4.80	4.20	4.00
	Raw fat %	5.00	5.10	5.20	5.50
	Raw fiber %	3.80	3.40	2.70	2.60
	Lysine %	1.34	1.21	1.12	1.10
	Calcium %	0.80	0.6	0.50	0.50
	Phosphorus %	0.52	0.45	0.37	0.34
	Methionine %	0.45	0.53	0.51	0.50
	Sodium %	0.15	0.15	0.15	0.15
Feed supplements	Vitamin D/ 25-hydroxycholecalciferol (IU/kg)	1000.00	1000.00	0.00	0.00
	Vitamin D3 (IU/kg)	3000.00	3000.00	3000.00	3000.00
	Vitamin A (IU/kg)	13000.00	10000.00	10000.00	10000.00
	Vitamin E (All-rac-alpha-tocopheryl acetate) (mg/kg)	80.00	60.00	30.00	30.00
	Iron-Fe (Ferrous sulfate, monohydrate) (mg/kg)	20.00	20.00	20.00	20.00
	Coated, granulated anhydrous calcium iodate, Iodine (mg/kg)	1.00	1.00	1.00	1.00
	Copper-Cu (Copper sulfate pentahydrate) (mg/kg)	8.00	8.00	8.00	8.00
	Copper-Cu (Copper trihydroxychloride) (mg/kg)	7.00	7.00	7.00	7.00
	Manganese-Mn (Manganese oxide(II)) (mg/kg)	80.00	80.00	80.00	80.00
	Zinc-Zn (Zinc sulfate, monohydrate) (mg/kg)	40.00	40.00	40.00	40.00
	Zinc-Zn (Zinc hydroxychloride monohydrate) (mg/kg)	35.00	35.00	35.00	35.00
	Sodium selenite, Selenium-Se (mg/kg)	0.30	0.30	0.30	0.30
	Guanidinoacetic acid (mg/kg)	0.00	573.00	573.00	573.00
Feed zootechnical additives	Endo-1,4-beta-xylanase (U/kg)	2428.00	2428.00	2428.00	2388.00
	Endo-1,3 (4)-beta-glucanase (U/kg)	302.00	302.00	302.00	0.00
	6-phytase (FTU/kg)	1493.00	1493.00	1990.00	1493.00
	Endo-1,4-beta-mannanase (U/kg)	52537.00	52537.00	52537.00	52537.00
	*Bacillus lichiniformis* (CFU/kg)	1.00×10^9	1.00×10^9	0.00	0.00

**Table 4:** Feed ingredients.

Starter	Grower 1	Grower 2	Finisher
Corn, soybean meal, wheat, sunflower meal, sunflower seeds, calcium carbonate, oat, rapeseed meal, animal fat (poultry), monocalcium phosphate, vegetable oils and fats (sunflower-crude, sodium chloride, animal fat (pork), sodium sulfate wheat bran, vegetable oils and fats (sunflower seeds)	Wheat, soybean meal, sunflower seeds, dehulled sunflower seeds, triticale, calcium carbonate, oat, wheat grain flour, calcium carbonate, vegetable oils and fats (sunflower-crude) monocalcium phosphate, animal fat (poultry), sodium chloride, sodium sulfate, wheat bran, vegetable oils and fats (sunflower seeds),flax seeds	Corn, wheat, soybean meal, calcium carbonate, sunflower seeds, dehulled sunflower seeds, potato processing products, animal fat (poultry), vegetable oils and fats (sunflower-crude), sodium chloride, sodium sulfate, monocalcium phosphate, wheat bran, vegetable oils and fats (sunflower seeds)	corn, wheat, soybean meal, potato processing products, calcium carbonate, vegetable oils and fats (sunflower-crude), animal fat (poultry), sodium chloride, monocalcium phosphate, rapeseed meal, calcium carbonate, wheat bran, vegetable oils and fats (sunflower seeds)

**Table 5:** Average Feed Intake of Ross 308 Broilers from day 1 to 41.

Age (Days)	Phase	Feed Intake (g/bird/day)	Cumulative Feed Intake (g/bird)
1-10	Starter	13-50	330-350
11-20	Grower 1	55-100	900-1050
21-30	Grower 2	110-150	1100-1300
31-41	Finisher	155-200	1300-1600
Total (1-41)	Overall	-	3630-4300

The other methods used are described below.

The macroscopic scoring scheme of breast myopathies was performed 24 hours after slaughter by three trained panellists. The scoring was performed according to the following scheme:

Spaghetti meat (SM)

SM0 (absent): without myofiber separation

SM1 (present): obvious myofiber separation

Woody breast (WB)

WB0 (absent): without any hardness or paleness areas

WB1 (moderate): moderate increase in hardness either in the cranial or caudal areas of the fillets or both

WB2 (severe): marked increase in hardness diffusely throughout the fillets

White striping (WS)

WS0 (absent): no distinct white lines

WS1 (mild): 1–40 white lines with thicknesses < 1 mm

WS2 (moderate): > 40 white lines, or 1–5 line(s) of 1.0 mm–1.9 mm in width

WS3 (severe): > 5 lines with a thickness of 1.0 mm to 1.9 mm or ≥ 1 line with a thickness of 2.0

The natural drip was measured via the EZ-Drip Loss method (Oksama 2004 a, b). Two 2.0-cm steaks were cut across the fibre direction, and one steak was cut from the below-middle position of each breast muscle, 48 hours postmortem. The samples for EZ-Drip loss were bored in the fibre direction in the middle of the piece of meat steak with a Ø 25 mm circular knife (1 sample per steak). First, the weight of the EZ-Drip loss container was recorded, and then the weight of the container with the bored meat sample was recorded. The EZ-Drip Loss samples were placed in a vertical fibre direction in the containers. The containers were firmly closed and stored in a metal holder at 4°C for 24 hours. After 24 hours, the samples were removed from the containers, and the containers with meat juice were reweighed.

The calculation of EZ-Drip loss was conducted according to the following formula:

EZ-Driploss = [(W1-Wc)/(Wt –Wc)] × 100 where:

Wc is the weight of the empty EZ-Drip Loss container

Wt is the weight of the EZ-Drip Loss container with meat and juice.

W1 is the weight of the EZ-Drip Loss container with juice.

The cooking loss of the thawed samples was measured for two months at –20°C, according to the modified method of Honikel (1998). The upper parts of the *pectoralis major* muscles were weighed and packed in polyethylene bags meant for the thermal processing of food (sous vide bags; 67 g/m^2^; density; oxygen permeability; ≤ 4.0 g/m^2^; ≤ 65 cm3/m^2^’d*bar/24 h water vapour permeability), with the bag’s wall firmly adhering to the meat sample. A calibrated thermocouple probe was inserted into the center of the additional sample. The samples were placed in a water bath preheated to 76°C and cooked until they reached an internal temperature of 72°C. The samples were removed from the water bath, immediately submerged in an ice bath, and reweighed (without a vacuum bag). Two methods of cooking loss measurement were applied.

Cooking loss –Samples were removed from the vacuum bags and placed in the refrigerator at 4°C. Then, the samples were reweighed after 12 hours. The weight loss was calculated via the following formula:

Cookingloss(%)=(weightlossaftercooking*100)/weightbeforecooking

The shear force measurements were performed with the BMORSE test (blunt blade) attached to the TA. XT Plus Texture Analyser (Stable 136 by MicroSystems, Warrington, UK). Immediately after cooking, the samples were cooled in an ice bath and placed in a refrigerator at 4°C. The BMORSE test was performed (6 h to 24 h after cooking) under the following test conditions: 5 kg.f (50 N) load cell; blunt blade (height 24.0 mm; width 8.0 mm; thickness 0.5 mm); 20 mm penetration depth; 10 mm/sec cross-head speed; 10 g trigger force; 6 shears per sample; and shearing the sample at 90° in the muscle fibre direction. Six subsamples (shears) were used per sample.

### Statistical analysis

The meat quality traits were examined in the R statistical package. The normal distribution of all the examined traits was confirmed with the Shapiro test. Then, a two-way ANOVA with interaction was performed as follows:

EXP12×2 experimental design with enrichment effects (access vs. no access to perches, treatment groups 1 and 2) and space allowance (stocking density of 35 kg/m^2^ vs. 41 kg/m^2^, treatment groups 3 and 4).

EXP22×2 Experimental design with the effect of enrichment (access vs. no access to perches, treatment groups 1 and 2) and access to an additional source of roughage (conventional feed vs conventional feed with dried lucerne, treatment groups 5 and 6).

The models were as follows:

y=enrichment+density+enrichment*density,

and

y=enrichment+roughage+enrichment*roughage,

where *y* is a vector of meat quality parameters; “enrichment” stands for the treatment groups with or without enrichment in the form of access to perches; “density” represents the treatment groups with and without reduced stocking density; and “roughage” stands for the treatment groups with and without access to dried lucerne. The “*” stands for interaction between the treatment groups.

The incidences of myopathies were examined as nominal traits:

1. Spaghetti meat (0-absent; 1-present);
2. Woody breast (0-absent; 1-moderate; 2-severe);
3. White striping (0- absent; 1-mild; 2-moderate; 3-severe).

The effects of space allowance and enrichment, as well as the effect of enrichment and roughage on the number of cases, were compared between groups with the Chi-square test.

## Results

The mean slaughter weight of the birds was 2580 g (± 41), with a mean carcass weight of 2050 g (± 30).

### Fresh meat (45 min–48 h from the slaughter)

Table 6 present the physicochemical traits of chicken breasts examined 45 min–48 h from the slaughter. In EXP1, the enrichment did not influence any meat quality traits. The stocking density was found to affect the b* index (P = 0.014), which was 4.22 % greater in DEN-41 compared to DEN-35 groups. No interactions between fixed factors were found. In EXP2, the enrichment did not influence meat quality attributes examined directly post-mortem. An effect of roughage was noted on the pH24, pH48, and b*, with highly significant interactions between factors noted for pH24 (P< 0.000), and b* (P< 0.000). The pH48 value was 0.01 % greater in LU compared to N-LU groups (P = 0.047).

**Table 6:** The effects of access to perches and different stocking densities (EXP1) as well as access to perches and to roughage (EXP2) on the physicochemical qualities of fresh chicken breast.

EXP1							p-values	SEM
Trait	PER	N-PER	DEN-35	DEN-41	EN	DEN	EN*DEN	
pH_45_	6.43	6.70	6.37	6.79	0.439	0.256	0.261	0.14
pH_24_	5.88	5.87	5.88	5.87	0.596	0.877	0.217	0.02
pH_48_	5.85	5.87	5.87	5.85	0.233	0.655	0.953	0.01
EZ-DripLoss%	0.64	0.58	0.63	0.59	0.479	0.655	0.954	0.05
L*	54.24	54.47	54.22	54.49	0.625	0.580	0.596	0.16
a*	-0.70	-0.49	-0.58	-0.60	0.065	0.839	0.867	0.06
b*	10.35	10.55	10.22	10.67	0.289	0.014	0.290	0.05
**EXP2**	**p-values**	**SEM**						
**Trait**	**PER**	**N-PER**	**N-LU**	**LU**	**EN**	**RO**	EN*RO	
pH_45_	6.44	6.76	6.79	6.44	0.344	0.359	0.348	0.11
pH_24_	5.90	5.89	5.87	5.92	0.52	<0.000	<0.000	0.01
pH_48_	5.88	5.89	5.85	5.91	0.839	0.047	0.420	0.01
EZ-DripLoss%	0.66	0.55	0.59	0.62	0.261	0.804	0.626	0.04
L*	53.78	54.42	54.49	53.71	0.155	0.087	0.700	0.18
a*	-0.66	-0.64	-0.60	-0.70	0.845	0.361	0.121	0.04
b*	10.36	10.51	10.67	10.21	0.4	0.001	<0.000	0.07

PER –access to perches; N-PER –no access to perches; DEN-35 –stocking density of 35 kg/m^2^; DEN-41 –no enrichment, stocking density of 41 kg/m^2^; N-LU –no access to dried lucerne; LU –access to dried Lucerne; EN –influence of enrichment: perches vs no perches; DEN –influence of 35 or 41 kg/m^2^ stocking density; RO –influence of roughage: dried lucerne vs no dried lucerne; EN*DEN –interaction between two factors; EN*RO –interaction between two factors

### Freezer-stored meat

Table 7 presents the results of the meat quality examinations performed on the freezer-stored samples, with no enrichment-caused variation in any of the examined traits. Stocking density influenced the pH after thawing, with the DEN-35 group showing a greater value than DEN-41 (by 0.67 %; 0.035). A significant interaction between the fixed factors was noted for the BMORS energy (P = 0.002) in EXP1. In EXP2, enrichment influenced multiple traits of freezer-stored meat, including thaw loss, cooking loss, and all the measurements within the Warner Bratzler and BMORS methods (Table 7). Significant interactions were found between enrichment and roughage for the pH measured on the freezer-stored samples, thaw loss, BMORS force and energy. Cooking loss was by 0.57 pp greater in birds with access to perches (P< 0.000) compared to N-PER group. Warner Bratzler textural parameters were generally greater in N-PER meat compared to PER. This covered the force (by 10.11 %; P< 0.000), energy (by 8.51 %; P< 0.000), and YM 20-80 % (by 0.90 pp; P< 0.000). Among the BMORS parameters, the YM 0-10 % was greater in the PER birds compared to N-PER by 0.07 pp (P< 0.000), with the reverse relation found for YM 20-80 %.

**Table 7:** Influence of access to perches and stocking density (EXP1) as well as access to perches and to roughage (EXP2) on the physicochemical qualities of frozen and thawed chicken breast.

EXP1	p-values	SEM						
Trait	PER	N-PER	DEN-35	DEN-41	EN	DEN	EN*DEN	
pH	5.90	5.91	5.93	5.89	0.407	0.035	0.244	0.01
Thaw loss %	8.03	7.86	7.57	8.28	0.618	0.052	0.966	0.11
Cooking loss %	14.30	13.94	13.98	14.28	0.889	0.529	0.263	0.20
Warner Bratzler								
Force N	16.59	17.06	17.29	16.36	0.511	0.194	0.647	0.36
Energy Nmm	166.11	179.74	177.01	168.80	0.086	0.292	0.097	3.21
YM 0-10 %	0.92	0.36	0.69	0.59	0.213	0.821	0.694	0.28
YM 20-80 %	2.68	2.54	2.60	2.62	0.566	0.228	0.582	0.23
BMORS								
Force N	8.94	8.97	8.82	9.11	0.954	0.639	0.458	0.26
Energy Nmm	118.32	117.61	113.17	123.11	0.924	0.182	0.002	2.94
YM 0-10 %	0.42	0.39	0.40	0.40	0.196	0.837	0.073	0.03
YM 20-80 %	0.69	0.68	0.72	0.65	0.946	0.316	0.162	0.01
**EXP2**	**p-values**	**SEM**						
**Trait**		**PER**	**N-PER**	**N-LU**	**LU**	**EN**	**RO**	EN*RO
pH	5.94	5.92	5.89	5.98	0.187	0.002	0.001	0.01
Thaw loss %	8.43	7.50	8.28	7.60	0.024	0.089	0.035	0.16
Cooking loss %	14.21	13.64	14.28	13.60	<0.000	0.176	0.231	0.25
	Warner Bratzler							
Force N	16.02	17.64	16.36	17.34	<0.000	0.232	0.311	0.31
Energy Nmm	163.60	178.83	168.80	173.99	<0.000	0.895	0.875	3.39
YM 0-10 %	0.70	1.25	0.59	1.37	0.017	0.154	0.102	0.23
YM 20-80 %	2.57	3.47	2.62	3.44	<0.000	0.166	0.156	0.25
	BMORS							
Force N	8.92	8.79	9.11	8.61	<0.000	0.003	0.001	0.22
Energy Nmm	132.38	113.42	123.11	121.99	<0.000	0.005	0.005	2.81
YM 0-10 %	0.44	0.37	0.40	0.40	<0.000	0.896	0.887	0.01
YM 20-80 %	0.60	0.68	0.65	0.63	<0.000	0.986	0.82	0.02

PER –access to perches; N-PER –no access to perches; DEN-35 –stocking density of 35 kg/m^2^; DEN-41 –no enrichment, stocking density of 41 kg/m^2^; N-LU –no access to dried lucerne; LU –access to dried Lucerne; EN –influence of enrichment: perches vs no perches; DEN –influence of 35 or 41 kg/m^2^ stocking density; RO –influence of roughage: dried lucerne vs no dried lucerne; EN*DEN –interaction between two factors; EN*RO –interaction between two factors; BMORS - Blunt Meullenet-Owens Razor Shear; YM –Young Modulus

### Basic chemical composition

Enrichment influenced the moisture and fat content in meat (Table 8). In EXP1, birds with access to perches had 0.92 percentage points (pp) greater (P = 0.011) content of moisture and 0.74 pp lower (P = 0.014) content of fat compared to N-PER group. Stocking density also caused a variation in fat content, and DEN-35 birds had 0.60 pp greater fat compared to the DEN-41 group (P 0.042). No interactions between the fixed factors were found. In EXP2, the PER birds’ meat was characterised with a greater content of moisture (by 0.96 pp; P< 0.000), and a lower content of fat (by 1.1 pp; P< 0.000) compared to N-PER.

**Table 8:** Influence of access to perches and stocking density (EXP1) as well as access to perches and to roughage (EXP2) on the proximal chemical composition of chicken breasts (%).

EXP1	p-values	SEM						
Trait	PER	N-PER	DEN-35	DEN-41	EN	DEN	EN*DEN	
Total moisture	74.43	73.51	73.93	74.02	0.011	0.785	0.228	0.14
Extracted fat	3.43	4.17	4.10	3.50	0.014	0.042	0.297	0.13
Crude protein	21.85	21.92	21.73	22.04	0.832	0.391	0.362	0.14
**EXP2**	**p-values**	**SEM**						
**Trait**		**PER**	**N-PER**	**NRO**	**RO**	**EN**	**RO**	EN*RO
Total moisture	74.28	73.32	74.02	73.58	<0.000	0.180	0.194	0.14
Extracted fat	2.98	4.08	3.50	3.56	<0.000	0.736	0.462	0.13
Crude protein	21.66	22.10	22.04	21.72	0.249	0.403	0.927	0.14

PER –access to perches; N-PER –no access to perches; DEN-35 –stocking density of 35 kg/m2; DEN-41 –no enrichment, stocking density of 41 kg/m2; N-LU –no access to dried lucerne; LU –access to dried Lucerne; EN –influence of enrichment: perches vs no perches; DEN –influence of 35 or 41 kg/m2 stocking density; RO –influence of roughage: dried lucerne vs no dried lucerne; EN*DEN –interaction between two factors; EN*RO –interaction between two factors

### Myopathies

Spaghetti meat (Table 9) and wooden breast (Table 10) incidence were not influenced by any of the examined factors. White striping showed significant variation caused by the access to perches in EXP2. The N-PER birds showed a greater number of mild-scored samples compared to PER birds Table 11.

**Table 9:** Influence of access to perches and stocking density (EXP1) as well as access to perches and to roughage (EXP2) on the percentage share of spaghetti meat (SM) in the *Pectoralis major*.

EXP1		EN		p-value	DEN		p-value
		N-PER (n=59)	PER (n=58)		DEN-42 (n=60)	DEN-35 (n=57)	
SM	No (n=94)	47	47	0.999	51	43	0.285
	Yes (n=23)	12	11		9	14	

EXP2		EN		p-value	RO		p-value
		N-PER (n=59)	PER (n=58)		LU (n=60)	N-LU (n=60)	
SM	No (n=96)	52	55	0.556	56	51	0.240
	Yes (n=13)	8	5		4	9

**Table 10:** Influence of access to perches and stocking density (EXP1) as well as access to perches and to roughage (EXP2) on the percentage share of wooden breast (WB) in the *Pectoralis major*.

EXP1		EN		p-value	DEN		p-value
		N-PER (n=60)	PER (n=58)		DEN-42 (n=61)	DEN-35 (n=57)	
WB	No (n=108)	28	26	0.829	30	24	0.735
	Yes - 1 (n=96)	23	25		23	25	
	Yes - 2 (n=32)	9	7		8	8	

EXP2		EN		p-value	RO		p-value
		N-PER (n=60)	PER (n=60)		LU (n=59)	N-LU (n=61)	
WB	No (n=118)	28	31	0.470	29	30	0.472
	Yes - 1 (n=98)	24	25		26	23	
	Yes- 2 (n=24)	8	4		4	8

**Table 11:** Influence of access to perches and stocking density (EXP1) as well as access to perches and to roughage (EXP2) on the percentage share of white striping (WS) in the *Pectoralis major*.

EXP1		EN		p-value	DEN		p-value
		N-PER (n=60)	PER (n=58)		DEN-42 (n=01)	DEN-35 (n=50)	
WS	No (n=89)	21	26	0.250	14	28	0.598
	Yes - 1 (n=122)	28	27		39	28	
	Yes - 2 (n=27)	11	5		7	4	

EXP2		EN		p-value	RO		p-value
		N-PER (n=61)	PER (n=57)		LU (n=59)	N-LU (n=61)	
WS	No (n=89)	23	24	0.026	19	23	0.463
	Yes - 1 (n=122)	31	24		36	31	
	Yes - 2 (n=27)	7	9		4	7	

## Discussion

### Influence stocking density on chicken meat quality

Although greater space allowances are often associated with increased bird activity and consequently tougher meat (Castellini et al., 2002; da Silva et al., 2017; Zaid et al., 2020), this relationship was not observed in studies on fast-growing chickens. Consistent with the existing literature (Goo et al., 2019; Thema et al., 2022; Tong et al., 2012; Weimer et al., 2022), our findings also indicate no effect of stocking density on the water-holding capacity (WHC) or texture of chicken breast (measured with the Warner Bratzler and BMORS method). Stocking density did not influence other traits examined in this study directly post-mortem, like pH_45_, pH_24_, pH_48_ and colour coordinates, which is consistent with results presented by other authors on pH values (Goo et al., 2019; Thema et al., 2022; Tong et al., 2012; Weimer et al., 2022) or breast meat color (Goo et al., 2019; Thema et al., 2022; Tong et al., 2012; Weimer et al., 2022).

Myopathies are another group of traits crucial from the perspective of both the producers and consumers of chicken meat. Wooden breasts (WB), white stripes (WP), and spaghetti meat (SM) are commonly found in the *pectoralis major* of fast-growing chicken broilers (Petracci et al., 2019; Soglia et al., 2021; Wang et al., 2023). Notably, we had not found any variation in the incidence of myopathies linked to stocking density.

### Influences of access to perches on meat quality

While perches have been associated with improved welfare and increased physical activity in broilers (Bailie et al., 2013; Riber et al., 2018), their direct impact on meat quality remains unclear. One reason for this could be the limited degree of activity that perch promotes in fast-growing broilers. Unlike slow-growing or dual-purpose breeds, commercial broilers such as Ross 308 prioritize muscle accretion over activity, meaning that the presence of perches may not sufficiently stimulate increased exercise. Broilers tend to perch infrequently and prefer resting over perching as they age because of their rapid weight gain (Marchewka et al., 2023). This inactivity limits the potential benefits of perches on muscle structure, oxidative metabolism, and meat quality traits. According to the results of the presented study, access to perches had a different influence on meat quality, depending on the other factor used in the study (stocking density in EXP1 or access to roughage in EXP2). In EXP1, the access to perches did not influence any of the physicochemical traits (measured directly post-mortem and after frozen-storage). Still, it caused a significant variation in the elemental chemical composition. Some studies reported slight changes in L* and b* values when perches were provided (Aksit et al., 2017; Fidan et al., 2020), while others found no significant differences in the color parameters, similar to the findings in this study (Marchewka et al., 2023). In EXP2, access to perches increased moisture and reduced fat content in the chicken breast compared with groups with no enrichment in pens. The Warner Bratzler measurements showed greater force and energy of shear in the meat of birds from a non-enriched environment compared to those having access to perches. This means that the birds with perches in pens had more tender meat, which is a positive aspect from the perspectives of both consumers and producers. The BMORS measurements were influenced not only by access to perches but also by roughage access and the interaction between these two factors. These findings suggest that perches alone may not be a sufficient enrichment strategy to induce physiological or metabolic changes in broilers that would translate into altered meat quality. Other forms of enrichment, such as foraging material or access to outdoor areas, may have a more substantial influence on muscle function and, consequently, meat quality (Nasr et al., 2021). Ross 308 chickens may grow too rapidly to benefit from the provided enrichment fully, and their limited physical activity may not be sufficient to influence meat quality attributes (Güz et al., 2021). Interestingly, this study shows that when access to perches is combined to stocking density, it is neutral for the incidence of myopathies. While the combination of access to perches with access to roughage results with a positive impact of enrichment on chicken breasts. A decrease in the incidence of the mild spaghetti meat samples was noted in the PER groups compared to N-PER birds.

### Influence of access to roughage on meat quality

Dietary fibre has a beneficial influence on gastrointestinal tract development and physiology (Jha et al., 2021). Lucerne is a commercially available fibre source that is not only rich in protein but also contains bioactive compounds such as xanthophylls, β-carotene, flavonoids, polysaccharides, and saponins (Zheng et al., 2019). Lucerne supplementation may alter the color of meat, as it is a rich source of β-carotene and ɑ-tocopherol (Englmaierová et al., 2020). In the study of He et al. (2021), the b* value linearly increased with increasing lucerne supplementation. An effect of roughage supplementation on the yellowness of chicken meat was also noted in the presented study (P = 0.004), though no clear pattern of changed could be observed due to the interaction between the fixed factors (P = 0.001). Beyond yellowness, access to roughage influenced pH_24_, pH after thawing, and some textural measurements, though in all cases a significant interaction between enrichment and roughage access was also reported. No influence of access to roughage to the incidence of myopathies was found. These findings suggest that access to lucerne may result in substantial changes in chicken meat quality, but in combination with other husbandry factors.

### Study limitations

Several limitations should be acknowledged when interpreting the findings of this study. 1. The experiments were conducted exclusively on fast-growing commercial Ross 308 broilers. Results may therefore not be generalizable to slow-growing or alternative genotypes that differ in activity levels, growth rates, and muscle metabolism. 2. The study focused on physicochemical traits and the incidence of myopathies. Functional properties of meat (e.g., sensory evaluation, oxidative stability, or consumer acceptability tests) were not assessed, which could provide additional insight into quality outcomes. 3. Although perch access and space allowance were hypothesized to increase activity, direct behavioral or physiological measures of exercise intensity (e.g., locomotion tracking, metabolic markers) were not systematically quantified, limiting the ability to link enrichment use with muscle development.

## Conclusions

The findings indicate that stocking density had negligible effects on broiler breast meat quality. In contrast, environmental enrichment played a less or more significant role depending on the other factor included in the experiment. In EXP1, no consistent interactions between enrichment and stocking density were found, suggesting their effects were largely independent. In EXP2, access to perches and roughage influenced color traits, pH values, water-holding capacity, shear force, and chemical composition of the meat. Results from EXP2 demonstrated more pronounced and complex effects of enrichment and roughage, particularly after freezer storage. Enriched environments generally improved moisture retention and reduced fat content, while also modifying textural properties. Enrichment was also found to have a negative impact on myopathies when is combined with access to roughage. Overall, environmental enrichment enhanced several desirable quality attributes of broiler meat, highlighting its potential as a practical strategy to improve product quality without compromising bird health.

In commercial strains such as Ross 308, rapid growth and limited activity likely negate potential benefits from increased space or basic enrichment.

The lack of significant interactions between stocking density and perch enrichment, as well as the minimal effects of individual interventions, suggests the need for more impactful strategies—possibly involving dietary changes, genetic selection, or more engaging forms of enrichment—to influence meat quality and broiler welfare meaningfully.

Collectively, these findings indicate that environmental modifications alone have limited potential to enhance meat quality in fast-growing broilers, underscoring the need for integrated strategies that address both welfare and production efficiency.

### Availability of data and materials

The data obtained and analysed during the current study are available from the corresponding author on reasonable request.

## Funding

This study was conducted within the project entitled “Linking extensive husbandry practices to the intrinsic quality of pork and broiler meat” - mEATquality, funded by the European Union’s Horizon 2020 Research and Innovation Program under Grant Agreement No 101000344.

## CRediT authorship contribution statement

**Agnieszka Ludwiczak:** Conceptualization, Data curation, Formal analysis, Investigation, Methodology, Software, Visualization, Writing – original draft, Writing – review & editing. **Patryk Sztandarski:** Conceptualization, Data curation, Methodology, Resources, Writing – original draft, Writing – review & editing. **Joanna Składanowska-Baryza:** Data curation, Investigation, Writing – original draft. **Karolina Szulc:** Data curation. **Gabriela Cieleń:** Data curation, Software. **Aneta Jaszczyk:** Conceptualization, Data curation. **Magdalena Solka:** Conceptualization, Data curation. **Grzegorz Pogorzelski:** Conceptualization, Data curation. **Jarosław O. Horbańczuk:** Conceptualization, Data curation. **Joanna Marchewka:** Conceptualization, Funding acquisition, Investigation, Methodology, Project administration, Resources, Writing – original draft, Writing – review & editing. **Ewa Sell-Kubiak:** Formal analysis, Funding acquisition, Methodology, Project administration, Resources, Software, Supervision, Writing – original draft, Writing – review & editing.

## Disclosures

The authors declare no conflicts of interest.
